# CCR5Δ32 Polymorphism and Inflammatory Bowel Disease: A Case–Control and Genotype–Phenotype Study in a Polish Population

**DOI:** 10.3390/ijms27188297

**Published:** 2026-09-17

**Authors:** Pawel Petryszyn, Klaudia Koj, Robert Dudkowiak, Agnieszka Gruca, Elzbieta Poniewierka, Krystyna Głowacka

**Affiliations:** 1 Department of Clinical Pharmacology, Wroclaw Medical University, Borowska 211A, 50-556 Wroclaw, Poland; 2 Department of Gastroenterology and Internal Medicine, University Clinical Center, Medical University of Warsaw, Banacha 1A, 02-097 Warsaw, Poland; 3 Department of Gastroenterology, Hepatology and Internal Medicine, Wroclaw Medical University, Borowska 213, 50-556 Wroclaw, Poland

**Keywords:** CCR5, CCR5Δ32, inflammatory bowel disease, ulcerative colitis, Crohn’s disease, chemokine receptor, genetic polymorphism, leukocyte trafficking, sex differences

## Abstract

C-C chemokine receptor 5 (CCR5) contributes to leukocyte trafficking and intestinal inflammation, whereas the functional CCR5Δ32 deletion markedly impairs receptor expression. Its role in inflammatory bowel disease (IBD) susceptibility remains uncertain. We evaluated CCR5Δ32 in 274 patients with IBD, including 141 with Crohn’s disease (CD) and 133 with ulcerative colitis (UC), and 100 controls using polymerase chain reaction genotyping; phenotype-related associations were explored in a clinically characterized subgroup. Overall CCR5 genotype distributions did not differ between patients with IBD and controls (*p* = 0.11). In the dominant model, CCR5Δ32 carriage was not associated with IBD overall (OR = 0.67, 95% CI 0.39–1.14; nominal *p* = 0.139; Holm-adjusted *p* = 0.319), CD, or UC. The CCR5Δ32 allele was nominally less frequent in UC than in controls (9.0% vs. 15.0%; OR = 0.56, 95% CI 0.317–0.995; *p* = 0.046), but this exploratory allele-level signal was not supported by the dominant model. CCR5Δ32 carriage was more frequent in men than women with IBD (OR = 2.07, 95% CI 1.06–4.03; *p* = 0.031), although the carrier-by-sex interaction for IBD susceptibility was not significant (*p* = 0.441). Direct within-disease phenotype comparisons were also non-significant; the strongest signal was lower carriage in corticosteroid-exposed versus unexposed UC (OR = 0.26, 95% CI 0.05–1.28; *p* = 0.065). CCR5Δ32 does not appear to be a major IBD susceptibility variant, but the UC-, sex-, and phenotype-related observations warrant independent replication.

## 1. Introduction

Inflammatory bowel disease (IBD), comprising Crohn’s disease (CD) and ulcerative colitis (UC), includes chronic, relapsing inflammatory disorders of the gastrointestinal tract that impose a substantial lifelong clinical burden. The epidemiology of IBD has changed considerably over recent decades: the highest prevalence remains in Europe and North America, while incidence has increased markedly in newly industrialized regions, making IBD an increasingly global disease [1]. Global Burden of Disease 2021 estimates likewise show a continuing increase in the absolute number of affected individuals worldwide [2].

Current models consider IBD to result from a complex interaction between genetic susceptibility, environmental exposures, intestinal microbiota, epithelial barrier dysfunction, and dysregulation of innate and adaptive immune responses. Genetic studies have identified numerous susceptibility loci involving microbial sensing, autophagy, epithelial homeostasis, and cytokine signalling. Among the best-established examples are *NOD2*, *ATG16L1*, and *IL23R*, which helped establish the roles of bacterial sensing, autophagy, and the IL-23 pathway in IBD pathogenesis [3,4,5]. Large-scale genetic studies have additionally implicated pathways regulating leukocyte adhesion and trafficking, including integrin-related loci [6].

Leukocyte recruitment into the intestinal mucosa is a particularly important component of chronic intestinal inflammation, as also demonstrated by the therapeutic efficacy of α4β7-integrin blockade in UC and CD [7,8]. Chemokines provide an additional level of regulation by directing the migration and positioning of selected leukocyte populations. C-C chemokine receptor 5 (CCR5) is a seven-transmembrane G-protein-coupled receptor that mediates responses to several inflammatory chemokines, particularly CCL3, CCL4, and CCL5/RANTES [9]. In activated CD4-positive T cells, CCL5-mediated chemotaxis involves PI3K- and mTOR-dependent signalling and regulation of the mTOR/4E-BP1 translational pathway, linking CCR5 activation directly to cellular migration [10].

Several lines of evidence implicate the CCL5/CCR5 axis in intestinal immune-cell trafficking. Human intestinal lamina propria and intraepithelial lymphocytes express functional CCR5, together with other inflammation-associated chemokine receptors, and migrate toward their respective ligands [11]. In actively inflamed IBD mucosa, CCL5 and other chemokines are produced by macrophages, T lymphocytes, and endothelial cells, with greater expression in more severely inflamed tissue [12]. In UC, increased numbers of CCR5-positive cells have been demonstrated in inflamed colonic mucosa and were related to disease activity [13]. In CD, CCR5- and CXCR3-positive T cells accumulate in inflamed mucosa and around CCL5-expressing non-caseating granulomas, providing a spatial link between chemokine production and recruitment of effector T cells [14]. Furthermore, mucosal CCR5 expression has been reported to be higher in active IBD than in remission and healthy controls and to correlate with lymphocytic infiltration [15].

Experimental evidence provides additional support for a functional contribution of CCR5 to intestinal inflammation. In several murine colitis models, genetic ablation of CCR5 reduced recruitment and activation of CCR5-bearing CD4-positive and CD11b-positive leukocytes in the intestinal mucosa, while pharmacological blockade with maraviroc attenuated inflammation by selectively reducing CCR5-dependent leukocyte trafficking [16]. Other chemokine pathways have also been investigated in IBD, illustrating that immune-cell positioning is a broader pathogenic and therapeutic theme rather than a mechanism specific to CCR5. For example, the gut-homing CCR9/CCL25 pathway progressed to phase 3 therapeutic development in CD, although the CCR9 antagonist vercirnon ultimately failed to demonstrate efficacy as induction therapy [17].

The biological relevance of CCR5 is strengthened by the existence of the naturally occurring CCR5Δ32 variant. This 32-base-pair deletion within the coding region causes a frameshift and produces a truncated, nonfunctional receptor with markedly impaired cell-surface expression. The variant occurs predominantly in populations of European ancestry, and homozygosity confers strong protection against infection with CCR5-tropic HIV-1 [18]. This naturally occurring loss-of-function phenotype provided compelling human target validation for CCR5 inhibition and contributed to the development of maraviroc, an allosteric CCR5 antagonist with established clinical efficacy in CCR5-tropic HIV infection [19]. The consequences of altered CCR5 signalling extend beyond HIV: experimental work in stroke and traumatic brain injury, together with a clinical stroke cohort, implicates CCR5 in neurological recovery [20], while the CCL5/CCR5 axis has also been linked to immune-cell recruitment, tumour progression, and metastasis in several malignancies [21].

Given its role in leukocyte migration, CCR5 represents a biologically plausible modifier of chronic immune-mediated diseases, including IBD. Nevertheless, previous studies evaluating CCR5Δ32 as an inherited susceptibility factor for IBD have generally not established a reproducible association with overall IBD, CD, or UC susceptibility [22,23]. Studies of CCR5 and related chemokine-receptor polymorphisms have occasionally suggested relationships with particular clinical phenotypes or disease activity rather than with overall disease risk, but these observations remain inconsistent [22,24]. Many of the available studies were performed in relatively small or geographically restricted populations, and detailed genotype-phenotype analyses remain limited. The present study was deliberately focused on CCR5Δ32 as a biologically well-characterized loss-of-function variant rather than on comprehensive genetic variation across the CCR5 locus. We therefore aimed to determine the frequencies of CCR5Δ32 genotypes and alleles in patients with IBD and controls, to evaluate their associations with susceptibility to IBD and its major subtypes, CD and UC, and to explore potential relationships between CCR5Δ32 and selected demographic and clinical characteristics and disease phenotypes.

## 2. Results

### 2.1. Demographic and Clinical Characteristics

A total of 274 patients with inflammatory bowel disease (IBD) and 100 controls were included in the genetic association analysis. The IBD group comprised 164 men and 110 women; among patients with available age data, the mean age was 38.0 ± 15.0 years. The control group consisted of 41 men and 59 women, with a mean age of 52.5 ± 20.1 years.

Detailed clinical and phenotypic data were available for a subgroup of 100 patients with IBD, comprising 50 patients with Crohn’s disease (CD) and 50 with ulcerative colitis (UC). Their characteristics are summarized in Table 1. Compared with the remaining 174 patients, the clinically characterized subgroup did not differ significantly in sex distribution (57.0% vs. 61.5% male; *p* = 0.522), CD/UC composition (50.0% vs. 52.3% CD; *p* = 0.802), or CCR5Δ32 carrier status (24.0% vs. 16.1%; *p* = 0.113). The mean age at diagnosis was 27.6 ± 13.9 years in CD and 29.3 ± 13.3 years in UC. Ileocolonic involvement was the most common CD location, while stricturing disease was the predominant behaviour. Among patients with UC, extensive colitis was the most frequent disease extent. Most patients had been exposed to 5-aminosalicylic acid (5-ASA), and substantial proportions had received corticosteroids or immunosuppressive treatment.

### 2.2. CCR5 Polymorphism and Disease Susceptibility

Genotype distributions were consistent with Hardy–Weinberg equilibrium in both controls and patients with IBD (*p* = 0.225 and *p* = 0.742, respectively).

The overall distribution of CCR5 genotypes did not differ significantly between patients with IBD and controls (*p* = 0.11), nor when CD (*p* = 0.36) and UC (*p* = 0.12) were analyzed separately. Genotype and allele frequencies and their associations with disease susceptibility are summarized in Table 2.

In the dominant model, CCR5Δ32 carriage was not significantly associated with IBD overall (OR = 0.67, 95% CI 0.39–1.14; nominal *p* = 0.139; Holm-adjusted *p* = 0.319), CD (OR = 0.74, 95% CI 0.40–1.35; nominal *p* = 0.322; Holm-adjusted *p* = 0.322), or UC (OR = 0.60, 95% CI 0.32–1.12; nominal *p* = 0.106; Holm-adjusted *p* = 0.319). For IBD overall, the sex-adjusted model yielded a similar inverse estimate that remained non-significant (sex-adjusted OR = 0.60, 95% CI 0.34–1.04; *p* = 0.066). Age-adjusted case–control regression could not be performed because individual-level age data were unavailable for controls. At the allele level, CCR5Δ32 was present in 10.0% of alleles in patients with IBD compared with 15.0% in controls (OR = 0.63, 95% CI 0.392–1.019; nominal *p* = 0.058). In UC, the allele was nominally less frequent than in controls (9.0% vs. 15.0%; OR = 0.56, 95% CI 0.317–0.995; nominal *p* = 0.046), whereas no corresponding association was observed for CD (OR = 0.70, 95% CI 0.408–1.199; nominal *p* = 0.192). Because the UC allele-level result arose from an exploratory analysis and was not supported by the dominant model, it was interpreted as hypothesis-generating rather than definitive evidence of association. At 80% power and α = 0.05, the overall dominant comparison could detect an odds ratio of approximately ≥2.01 or ≤0.43; substantially larger effects would be required for rare CCR5Δ32 homozygosity and small phenotype strata.

### 2.3. Association Between CCR5Δ32 and Sex

An exploratory sex-related difference in CCR5Δ32 frequency was observed within the IBD cohort. At least one CCR5Δ32 allele was present in 38 of 164 men (23.2%) compared with 14 of 110 women (12.7%), corresponding to an approximately twofold higher probability of variant carriage in men (OR = 2.07, 95% CI 1.06–4.03; *p* = 0.031). In sex-stratified case–control analyses, the carrier association was OR = 0.73 (95% CI 0.34–1.57; *p* = 0.421) in men and OR = 0.47 (95% CI 0.21–1.07; *p* = 0.083) in women.

At the allele level, CCR5Δ32 accounted for 12.2% of alleles in men and 6.8% in women (OR = 1.90, 95% CI 1.02–3.53; *p* = 0.040). No significant sex-related differences were observed in controls, either for CCR5Δ32 carriage (*p* = 0.53) or allele frequency (*p* = 0.49).

### 2.4. Phenotype-Related Analyses

The relationship between CCR5Δ32 and clinical phenotype was evaluated by direct within-disease comparisons of CCR5Δ32 carrier status between patients with and without each clinical characteristic within CD and within UC (Table 3). In CD, analyses included age at diagnosis, disease duration, disease location and behaviour, treatment exposure, and previous IBD-related surgery. In UC, age at diagnosis, disease duration, disease extent, immunosuppressant therapy, and corticosteroid exposure were assessed. For completeness, exploratory unadjusted comparisons of phenotype-defined patient subgroups with healthy controls are provided in Supplementary Table S1 and were not used to infer genotype–phenotype associations.

No statistically significant within-disease association was identified for any of the analyzed CD or UC phenotypes (Table 3). In UC, the strongest within-disease signal concerned corticosteroid exposure: CCR5Δ32 carriage was present in 5 of 35 corticosteroid-exposed patients (14.3%) and 6 of 15 unexposed patients (40.0%; OR = 0.26, 95% CI 0.05–1.28; nominal *p* = 0.065). All phenotype-related analyses were exploratory.

## 3. Discussion

The present study evaluated the CCR5Δ32 loss-of-function variant in a Polish IBD cohort and explored its relationship with selected clinical phenotypes. Four findings merit particular attention: (i) the overall genotype distribution and the dominant carrier model did not support a major association with IBD, CD, or UC; (ii) the CCR5Δ32 allele was nominally less frequent in IBD, but this exploratory signal was borderline and was not supported by the dominant model; (iii) CCR5Δ32 carriage and allele frequency were higher in men than women within the IBD cohort, but sex-stratified case–control analyses and the non-significant carrier-by-sex interaction did not support a sex-specific susceptibility effect; and (iv) direct within-disease phenotype analyses were negative, although corticosteroid-exposed UC showed a non-significant trend toward lower CCR5Δ32 carriage. Together, these findings argue against a large general susceptibility effect and support cautious, hypothesis-generating interpretation of the subtype-, sex-, and phenotype-related observations.

The absence of a strong overall association is broadly consistent with most previous studies. Rector et al. found no significant difference in CCR5Δ32 frequency between patients with IBD and controls and no preferential transmission of the variant in affected families [22]. Craggs et al. similarly found no association with CD or UC and no relationship with UC extent [23], while Martin et al. reported comparable genotype and allele distributions in CD, UC, and controls [25]. Herfarth et al. also found no significant association with CD susceptibility [26]. Of particular relevance, Central and Eastern European data point in a similar direction. In a Polish–Bosnian study, Adler et al. reported a lower CCR5Δ32 allele frequency in Polish patients with CD than in Polish controls, without a significant allele-level association; marked differences between the Polish and Bosnian cohorts additionally emphasized population heterogeneity [27]. A Czech–Slovak study by Hošek et al. likewise found no significant association of CCR5Δ32 with CD or UC, despite detecting associations for other IBD-related variants, including *NOD2* and *ICAM1* [28]. Thus, our negative result for CD is consistent with both the broader European literature and the limited evidence from neighbouring populations.

The nominally lower CCR5Δ32 allele frequency in UC (OR = 0.56, 95% CI 0.317–0.995; *p* = 0.046) should be interpreted cautiously. The confidence interval lay very close to the null value, the result arose among several exploratory comparisons, and the corresponding dominant carrier model was not significant. A similar direction of effect, although not statistically significant, was observed by Gorgi et al., who reported lower representation of CCR5Δ32 among Tunisian patients with UC [24], whereas other European studies found similar or numerically higher frequencies in UC [22,23,25]. Such heterogeneity may reflect differences in ancestry, control selection, sample size, or clinical composition. CCR5Δ32 itself shows substantial geographical variation in Europe [18,27], making careful population matching particularly important when the expected genetic effect is modest.

The direction of the UC observation is mechanistically plausible. CCR5 contributes to the recruitment of activated T cells and mononuclear cells to inflammatory sites, and increased numbers of CCR5-positive cells and enhanced CCR5 expression have been demonstrated in actively inflamed intestinal mucosa [13,14,15]. Experimental CCR5 deficiency or pharmacological blockade reduces mucosal recruitment of inflammatory leukocytes and attenuates colitis [16]. Because CCR5Δ32 markedly impairs functional cell-surface CCR5 expression [18], a lower allele frequency among patients with UC is compatible with the hypothesis that preservation of functional CCR5 signalling could favour CCR5-dependent leukocyte trafficking and may contribute to UC susceptibility. However, the modest effect size, borderline precision, lack of support from the dominant model, and exploratory analytical context preclude a causal or protective interpretation.

The phenotype-related analyses were predominantly negative. Direct within-disease comparisons did not identify a statistically significant association with age at diagnosis, disease duration, CD location or behaviour, UC extent, immunosuppressant or anti-TNF exposure, or IBD-related surgery. This is concordant with Rector et al., who found no relationship with age at diagnosis, localization, or surgery, although CCR5Δ32 homozygosity was associated with anal involvement in a very small subgroup [22]. Herfarth et al. likewise found no statistically significant phenotype associations, despite trends toward less upper gastrointestinal involvement and more stricturing disease among CCR5Δ32 carriers [26].

The corticosteroid-related observation may nevertheless warrant further investigation. In the within-UC comparison, CCR5Δ32 carriage was lower in corticosteroid-exposed than unexposed patients (14.3% vs. 40.0%; OR = 0.26, 95% CI 0.05–1.28; *p* = 0.065). Chemokine expression is closely linked to inflammatory activity in UC, and corticosteroids interfere with this network. Yang et al. demonstrated increased mucosal expression of multiple C-C and C-X-C chemokines in active UC and showed that dexamethasone downregulated several of these mediators; MIP-1β and RANTES/CCL5 showed the same direction of response [29]. A lower prevalence of a loss-of-function CCR5 allele among steroid-exposed patients might therefore identify a subgroup in whom preserved chemokine-dependent trafficking contributes to a more inflammatory phenotype requiring corticosteroid therapy. However, treatment exposure is an indirect marker influenced by disease activity, treatment era, and prescribing practice, and the present study was not designed as a pharmacogenetic analysis. This signal is therefore best regarded as hypothesis-generating.

The within-case sex association is another noteworthy but exploratory observation. CCR5Δ32 carriage was approximately twice as frequent among men as among women with IBD (OR = 2.07, 95% CI 1.06–4.03; *p* = 0.031), and the allele-level analysis showed a similar pattern (OR = 1.90, 95% CI 1.02–3.53; *p* = 0.040). However, sex-stratified case–control estimates were not statistically significant in men or women, and the formal carrier-by-sex interaction was also non-significant (*p* = 0.441). Thus, the within-case difference does not establish sex-specific susceptibility. Sex-dependent genetic effects are nevertheless increasingly recognized in IBD. In a large sex-stratified meta-analysis of more than 70,000 cases and controls, Khrom et al. identified genome-wide significant sex-dimorphic associations and sex-specific effects at established IBD loci [30]. At the individual-variant level, Liu et al. demonstrated a stronger association of the ATG16L1 rs3792106 variant with CD in women than in men; notably, part of this difference reflected sex-related allele-frequency differences among controls [31]. Conversely, Lin et al. reported a male-specific association of DLG5 R30Q with IBD, including both CD and UC [32]. Our CCR5 finding therefore remains of interest but requires replication in larger sex-stratified and ancestry-matched cohorts.

**Figure 1:**
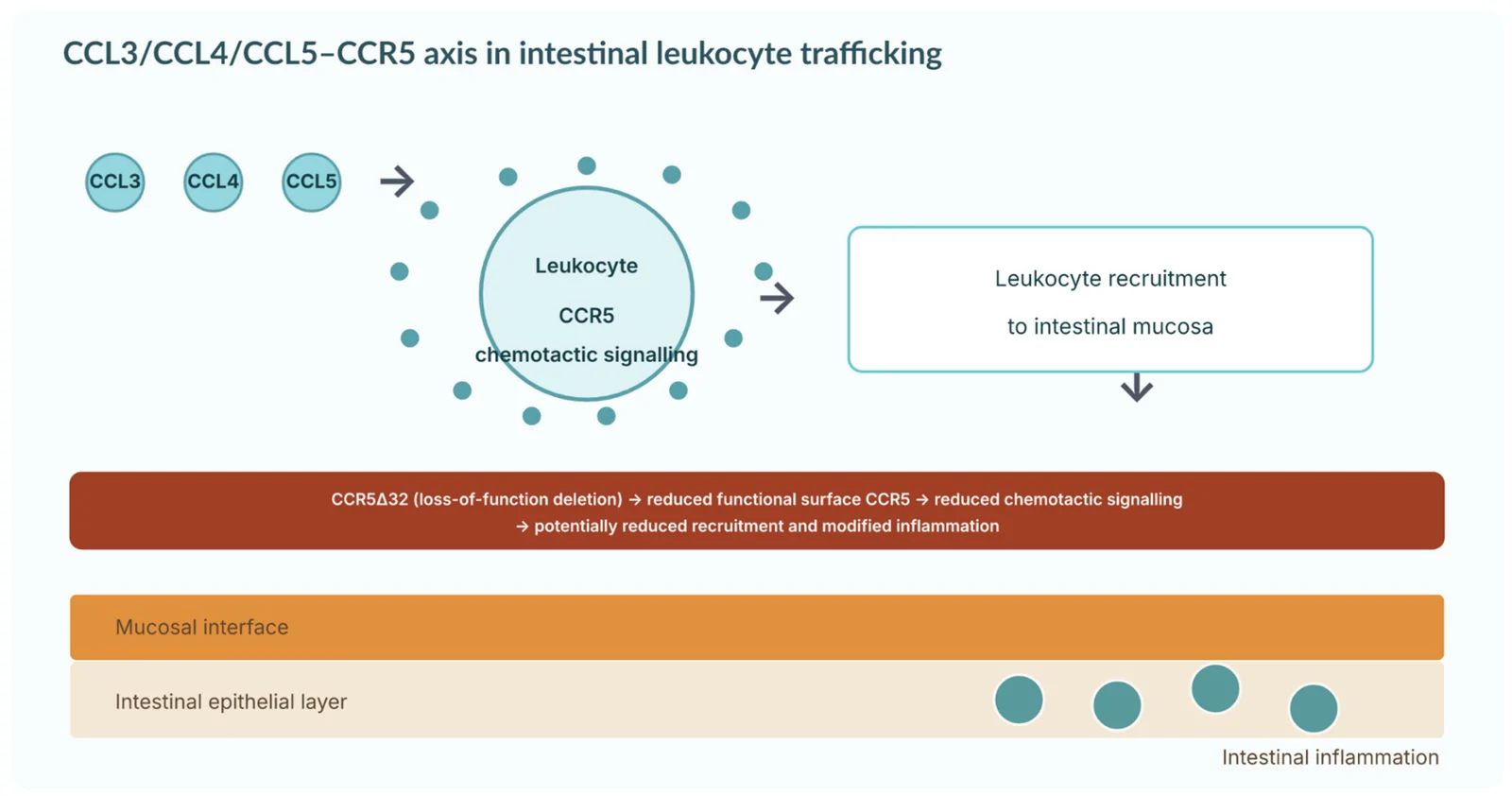
Illustrated conceptual model of CCL3/CCL4/CCL5 binding to CCR5 on leukocytes, intracellular chemotactic signalling, recruitment into intestinal mucosa, and inflammation. CCR5Δ32 produces a truncated, nonfunctional receptor with markedly impaired surface expression; reduced functional CCR5 expression has been reported in heterozygotes. The CCR5Δ32 panel is a hypothesis-generating model, not evidence of a causal effect on IBD susceptibility.

Figure 1 provides a conceptual framework for the biological plausibility of CCR5Δ32 in intestinal inflammation. CCL3, CCL4, and CCL5 activate CCR5 on leukocytes, promoting chemotactic signalling and recruitment to the intestinal mucosa [9,11,12,13,14,15,16]. CCR5Δ32 produces a frameshifted, C-terminally truncated receptor that does not undergo normal cell-surface trafficking; in heterozygous cells, reduced CCR5 expression appears to result mainly from reduced gene dosage, although retention of wild-type CCR5 through heterocomplex formation with the truncated receptor has also been proposed, suggesting context-dependent effects [33,34]. CCR5 nevertheless functions within overlapping leukocyte-migration and inflammatory signalling networks rather than as an isolated determinant; nitric oxide signalling, for example, modulates neutrophil adhesion, chemotaxis, respiratory burst, and other effector functions [35]. Thus, reduced CCR5 availability could modify leukocyte recruitment, but the schematic should be interpreted as a hypothesis-generating biological model rather than evidence that CCR5Δ32 causally alters IBD susceptibility.

The strengths of this study include a relatively large, clinically defined IBD cohort from a single geographical region, separate analyses of CD and UC, assessment of genotype, allele, and carrier frequencies, and genotype distributions consistent with Hardy–Weinberg equilibrium. Detailed phenotypic information in a subgroup additionally permitted assessment of Montreal disease characteristics, treatment exposure, and surgery. The study also expands the limited evidence on CCR5Δ32 in Polish and Central/Eastern European IBD populations.

Several limitations should be considered. The single-centre design and differences in age and sex distribution between patients and controls may introduce residual cohort effects. Because individual-level age data were unavailable for controls, age adjustment could not be performed and residual confounding by age cannot be excluded. Controls were recruited from the Lower Silesia region to match the geographical recruitment area, but genetic ancestry was not characterized using ancestry-informative markers. CCR5Δ32 homozygosity was uncommon, and the minimum detectable effect analysis confirmed limited precision for recessive and small phenotype-stratum analyses. Detailed clinical information was available only for a subset selected on the basis of sufficiently complete phenotype data, although this subgroup did not differ significantly from the remaining cohort in sex, disease subtype, or carrier status. Treatment variables reflected exposure rather than prospectively assessed therapeutic response. The study was deliberately focused on CCR5Δ32 and was not designed as a comprehensive analysis of the CCR5 locus; other functional or regulatory CCR5 variants, neighbouring chemokine-receptor genes, haplotypes, and gene–gene interactions were not evaluated. Finally, analyses beyond the dominant susceptibility model were exploratory, and the nominal UC, corticosteroid, and within-case sex observations should consequently be interpreted cautiously.

In conclusion, the present study supports previous evidence that CCR5Δ32 is not a major determinant of overall IBD susceptibility or of the principal clinical phenotypes of CD and UC. The nominally lower CCR5Δ32 allele frequency in UC was not supported by the dominant carrier model, and formal interaction analysis did not support a sex-specific susceptibility effect. The corticosteroid-related observation also remains exploratory. Replication in larger, ancestry-matched cohorts with prospectively collected phenotype and treatment data is needed. Future studies integrating CCR5Δ32 with other chemokine-pathway variants, sex-stratified analyses, and functional assessment of the CCL5/CCR5 axis in intestinal tissue may clarify whether CCR5 variation contributes to specific biological subtypes of IBD and whether this pathway has value for patient stratification or therapeutic targeting.

## 4. Materials and Methods

### 4.1. Patients and Controls

This case–control study evaluated the association between the CCR5Δ32 polymorphism and susceptibility to inflammatory bowel disease. The study included 274 patients with IBD, comprising 141 patients with Crohn’s disease and 133 patients with ulcerative colitis, recruited from the Department of Gastroenterology and Hepatology of Wroclaw Medical University, Poland, between 2019 and 2020. The diagnoses of CD and UC were established by a gastroenterologist based on clinical assessment and endoscopic, radiological, and/or histopathological findings, in accordance with established diagnostic criteria.

The control group consisted of 100 participants without a diagnosis of gastrointestinal disease at the time of blood collection and without a history of cancer or autoimmune disease. Controls were recruited from the Lower Silesia region to match the geographical recruitment area of the IBD cohort. Genetic ancestry was not assessed using ancestry-informative markers.

Detailed clinical and phenotypic analyses were restricted to 100 patients (50 CD and 50 UC) for whom sufficiently complete phenotype data were available. Disease location and extent were classified according to the Montreal framework. CD behaviour categories reflected current or previously documented behaviour; consequently, B2 and B3 were not mutually exclusive. Treatment variables represented previous or current exposure rather than prospectively assessed treatment response; corticosteroid exposure included systemic corticosteroids and/or budesonide.

The study was conducted according to the guidelines of the Declaration of Helsinki and approved by the Bioethics Committee of Wroclaw Medical University (No. KB 498/2019). A written informed consent form for participation was provided to all participants and signed prior to inclusion in the study.

### 4.2. Genotyping

Peripheral whole blood was collected into tubes containing K_3_EDTA and stored at −20 °C until analysis. Genomic DNA was isolated from peripheral blood leukocytes using the E.Z.N.A.^®^ Blood DNA Mini Kit (Omega Bio-Tek, Norcross, GA, USA) according to the manufacturer’s instructions. DNA concentration and purity were assessed using a NanoDrop 2000c spectrophotometer (Thermo Fisher Scientific, Wilmington, DE, USA) by measuring absorbance at 230, 260, and 280 nm.

The CCR5Δ32 polymorphism was identified using conventional polymerase chain reaction with primers synthesized by Oligo.pl (Warsaw, Poland) and flanking the 32-base-pair deletion:

- forward: 5′-CTTCATTACACCTGCAGCTCT-3′;
- reverse: 5′-CACAGCCCTGTCCCTCTTCTTC-3′.

PCR was performed using EmeraldAmp Max HS PCR Master Mix (2× Premix; Takara Bio Inc., Kusatsu, Shiga, Japan) in a peqSTAR 96 Universal Gradient thermocycler (PEQLAB Biotechnologie GmbH, Erlangen, Germany). The amplification consisted of initial denaturation at 96 °C for 5 min, followed by 40 cycles of denaturation at 96 °C for 30 s, primer annealing at 55 °C for 30 s, and extension at 72 °C for 30 s, with a final extension at 72 °C for 5 min.

Amplification products were separated by electrophoresis in a 3% agarose gel together with a DNA size marker (DNA Marker pUC/Msp I (A&A Biotechnology, Gdańsk, Poland) and visualized under ultraviolet light. A 183 bp product corresponded to the wild-type CCR5 allele, whereas a 151 bp product indicated the CCR5Δ32 allele. The CCR5/CCR5 genotype was identified by a single 183 bp band, the CCR5Δ32/CCR5Δ32 genotype by a single 151 bp band, and the heterozygous CCR5/CCR5Δ32 genotype by the presence of both bands. A representative agarose gel electrophoresis image is provided in Supplementary Figure S1. Genotyping was performed in duplicate, and genotype calling was performed by two blinded pharmacy students.

### 4.3. Statistical Analysis

Participant characteristics were evaluated using descriptive statistics. Continuous variables were expressed as mean ± SD, and categorical variables as numbers and percentages. Age was available for 250 of 274 patients with IBD (91.2%); missing age values were not imputed. Individual-level age data were not available in the archived control dataset; consequently, age-adjusted case–control regression could not be performed.

Genotype and allele frequencies were compared between patients with IBD and controls and, separately, between the CD and UC subgroups and controls. Pearson’s chi-square test was used for categorical comparisons, with Fisher’s exact test when cell counts were sparse. Associations between CCR5 genotypes or alleles and disease susceptibility were expressed as odds ratios with 95% confidence intervals and two-sided *p*-values. Carriage of at least one CCR5Δ32 allele versus CCR5/CCR5 homozygosity (dominant model) was designated the principal genetic model for the susceptibility analysis, with the overall IBD comparison considered primary and the CD- and UC-versus-control comparisons secondary.

The association between CCR5Δ32 and sex was assessed at the carrier and allele levels within the IBD cohort. Sex-stratified case–control analyses, a sex-adjusted logistic model for overall IBD susceptibility as a sensitivity analysis, and a CCR5Δ32-carrier-by-sex interaction model were additionally performed. Phenotype-related analyses were based on direct within-disease comparisons of CCR5Δ32 carrier status between patients with and without each phenotype, performed separately for CD and UC using Fisher’s exact test. For completeness, exploratory unadjusted comparisons of phenotype-defined patient subgroups with controls are provided in Supplementary Table S1 and were not used to infer genotype–phenotype associations.

Conformity of genotype distributions with Hardy–Weinberg equilibrium was assessed separately in the control and IBD groups using an exact test. The three dominant-model susceptibility comparisons (IBD, CD, and UC versus controls) were adjusted using the Holm method. Genotype-level, allele-level, sex-related, interaction, and phenotype-related analyses were considered exploratory. Minimum detectable effects were estimated using the normal approximation for two independent proportions, assuming 80% power, a two-sided α of 0.05, the observed control carrier prevalence of 26%, and the available case–control sample sizes. Statistical analyses were performed using R software, version 4.0.3 (R Foundation for Statistical Computing, Vienna, Austria).

## Figures and Tables

**Table 1:** Demographic and clinical characteristics of the phenotypically characterized IBD subgroup and controls.

Characteristic	CD (N = 50)	UC (N = 50)	Controls (N = 100)
Sex, M/F, n (%)	25/25 (50/50)	32/18 (64/36)	41/59 (41/59)
Mean age, years (±SD)	35.8 (±13.9)	38.5 (±15.0)	52.5 (±20.1)
Mean age at diagnosis, years (±SD)	27.6 (±13.9)	29.3 (±13.3)	—
Disease location (CD)			
L1, ileal	12 (24%)	—	—
L2, colonic	12 (24%)	—	—
L3, ileocolonic	26 (52%)	—	—
Disease behaviour (CD)			
B1, inflammatory	10 (20%)	—	—
B2, stricturing	35 (70%)	—	—
B3, penetrating	15 (30%)	—	—
Disease extent (UC)			
E1, ulcerative proctitis	—	2 (4%)	—
E2, left-sided colitis	—	16 (32%)	—
E3, extensive colitis	—	32 (64%)	—
Severe disease	—	13 (26%)	—
5-ASA exposure	48 (96%)	49 (98%)	—
Immunosuppressant therapy	31 (62%)	22 (44%)	—
Corticosteroid use	31 (62%)	35 (70%)	—
Anti-TNF therapy	12 (24%)	5 (10%)	—
IBD-related surgery	17 (34%)	7 (14%)	—

Abbreviations: CD, Crohn’s disease; UC, ulcerative colitis; IBD, inflammatory bowel disease; 5-ASA, 5-aminosalicylic acid; TNF, tumour necrosis factor. B2 and B3 categories reflect current or previous disease behaviour and are not mutually exclusive.

**Table 2:** CCR5 genotype and CCR5Δ32 allele frequencies in patients with IBD, CD and UC compared with controls.

CCR5 Variant	Controls	IBD	OR (95% CI)	*p*	UC	OR (95% CI)	*p*	CD	OR (95% CI)	*p*
CCR5/CCR5	74/100 (74.0%)	222/274 (81.0%)	1.50 (0.87–2.57)	0.139	110/133 (82.7%)	1.68 (0.89–3.17)	0.106	112/141 (79.4%)	1.36 (0.74–2.49)	0.322
CCR5/CCR5Δ32	22/100 (22.0%)	49/274 (17.9%)	0.77 (0.44–1.36)	0.369	22/133 (16.5%)	0.70 (0.36–1.36)	0.292	27/141 (19.1%)	0.84 (0.45–1.58)	0.588
CCR5Δ32/CCR5Δ32	4/100 (4.0%)	3/274 (1.1%)	0.27 (0.06–1.21)	0.086	1/133 (0.8%)	0.18 (0.02–1.65)	0.168	2/141 (1.4%)	0.35 (0.06–1.92)	0.236
CCR5Δ32 allele	30/200 (15.0%)	55/548 (10.0%)	0.63 (0.392–1.019)	0.058	24/266 (9.0%)	0.56 (0.317–0.995)	0.046	31/282 (11.0%)	0.70 (0.408–1.199)	0.192
CCR5Δ32 carriers	26/100 (26.0%)	52/274 (19.0%)	0.67 (0.39–1.14)	0.139	23/133 (17.3%)	0.60 (0.32–1.12)	0.106	29/141 (20.6%)	0.74 (0.40–1.35)	0.322

Values are n/N (%). Odds ratios compare each patient group with controls. For the individual genotype rows, ORs compare presence versus absence of the specified genotype. The CCR5Δ32 carrier row corresponds to the dominant model (at least one Δ32 allele versus CCR5/CCR5). OR, odds ratio; CI, confidence interval; IBD, inflammatory bowel disease; CD, Crohn’s disease; UC, ulcerative colitis.

**Table 3:** Within-disease associations between CCR5Δ32 carrier status and clinical phenotypes in the clinically characterized CD and UC subgroups.

Clinical Characteristic	Phenotype Present, Carriers/N (%)	Phenotype Absent, Carriers/N (%)	OR (95% CI)	*p*
Crohn’s disease				
Age at diagnosis ≤ 25 years	8/33 (24.2%)	5/17 (29.4%)	0.77 (0.18–3.68)	0.741
Disease duration ≥ 8 years	8/23 (34.8%)	5/27 (18.5%)	2.31 (0.54–10.86)	0.215
Ileal disease (L1)	3/12 (25.0%)	10/38 (26.3%)	0.94 (0.14–4.85)	1.000
Colonic disease (L2)	4/12 (33.3%)	9/38 (23.7%)	1.59 (0.28–7.89)	0.707
Ileocolonic disease (L3)	6/26 (23.1%)	7/24 (29.2%)	0.73 (0.17–3.11)	0.751
Inflammatory disease (B1)	2/10 (20.0%)	11/40 (27.5%)	0.66 (0.06–4.13)	1.000
Stricturing disease (B2)	9/35 (25.7%)	4/15 (26.7%)	0.95 (0.21–5.15)	1.000
Penetrating disease (B3)	4/15 (26.7%)	9/35 (25.7%)	1.05 (0.19–4.85)	1.000
Immunosuppressant therapy	10/31 (32.3%)	3/19 (15.8%)	2.50 (0.52–16.43)	0.320
Corticosteroid use	9/31 (29.0%)	4/19 (21.1%)	1.52 (0.34–8.04)	0.742
Anti-TNF therapy	3/12 (25.0%)	10/38 (26.3%)	0.94 (0.14–4.85)	1.000
IBD-related surgery	6/17 (35.3%)	7/33 (21.2%)	2.00 (0.44–8.89)	0.322
Ulcerative colitis				
Age at diagnosis ≤ 25 years	7/25 (28.0%)	4/25 (16.0%)	2.01 (0.43–10.97)	0.496
Disease duration ≥ 8 years	8/26 (30.8%)	3/24 (12.5%)	3.04 (0.61–20.49)	0.175
Left-sided colitis (E2)	3/16 (18.8%)	8/34 (23.5%)	0.75 (0.11–3.87)	1.000
Extensive colitis (E3)	7/32 (21.9%)	4/18 (22.2%)	0.98 (0.20–5.40)	1.000
Immunosuppressant therapy	4/22 (18.2%)	7/28 (25.0%)	0.67 (0.12–3.17)	0.734
Corticosteroid use	5/35 (14.3%)	6/15 (40.0%)	0.26 (0.05–1.28)	0.065

Each comparison is made between patients with and without the stated phenotype within the indicated disease subgroup. Carrier status was defined as at least one CCR5Δ32 allele. B2 and B3 reflect current or previously documented behaviour and are not mutually exclusive. OR, odds ratio; CI, confidence interval; CD, Crohn’s disease; UC, ulcerative colitis; TNF, tumour necrosis factor.

## Data Availability

The original contributions presented in this study are included in the article/Supplementary Material. Further inquiries can be directed to the corresponding author.

## Supplementary Materials

The following supporting information can be downloaded at: https://www.mdpi.com/article/10.3390/ijms27188297/s1.
