# Supplementary material for: CCR5Δ32 Polymorphism and Inflammatory Bowel Disease: A Case–Control and Genotype–Phenotype Study in a Polish Population
Source: Int J Mol Sci. 2026 Sep 17;27(18):8297. doi: 10.3390/ijms27188297 (PMC13607550; doi:10.3390/ijms27188297)
Supplement: Supplementary file 1 [file ijms-27-08297-s001.zip › CCR5_IBD_Supplementary Table_S1.pdf]

Supplementary Table S1. Exploratory unadjusted case-control comparisons of CCR5Δ32 frequencies in phenotype-defined subgroups of Crohn's disease and ulcerative colitis.

Clinical characteristic	N	CCR5Δ32 allele, n/N (%)	OR (95% CI)	p	CCR5Δ32 carriers, n/N (%)	OR (95% CI)	p
**Crohn's disease**							
Age at diagnosis ≤25 years	33	10/66 (15.2%)	1.01 (0.47–2.20)	1.000	8/33 (24.2%)	0.91 (0.37–2.27)	1.000
Disease duration ≥8 years	23	9/46 (19.6%)	1.38 (0.60–3.15)	0.502	8/23 (34.8%)	1.52 (0.58–3.99)	0.441
Ileal disease (L1)	12	4/24 (16.7%)	1.13 (0.36–3.55)	0.768	3/12 (25.0%)	0.95 (0.24–3.77)	1.000
Colonic disease (L2)	12	4/24 (16.7%)	1.13 (0.36–3.55)	0.768	4/12 (33.3%)	1.42 (0.40–5.12)	0.731
Ileocolonic disease (L3)	26	7/52 (13.5%)	0.88 (0.36–2.14)	1.000	6/26 (23.1%)	0.85 (0.31–2.36)	1.000
Inflammatory disease (B1)	10	2/20 (10.0%)	0.63 (0.14–2.85)	0.746	2/10 (20.0%)	0.71 (0.14–3.57)	1.000
Stricturing disease (B2)	35	11/70 (15.7%)	1.06 (0.50–2.24)	0.849	9/35 (25.7%)	0.99 (0.41–2.38)	1.000
Penetrating disease (B3)	15	4/30 (13.3%)	0.87 (0.28–2.68)	1.000	4/15 (26.7%)	1.03 (0.30–3.54)	1.000
Immunosuppressant therapy	31	12/62 (19.4%)	1.36 (0.65–2.85)	0.431	10/31 (32.3%)	1.36 (0.56–3.25)	0.498
Corticosteroid use	31	10/62 (16.1%)	1.09 (0.50–2.38)	0.841	9/31 (29.0%)	1.16 (0.48–2.85)	0.817
Anti-TNF therapy	12	4/24 (16.7%)	1.13 (0.36–3.55)	0.768	3/12 (25.0%)	0.95 (0.24–3.77)	1.000
IBD-related surgery	17	6/34 (17.6%)	1.21 (0.46–3.18)	0.797	6/17 (35.3%)	1.55 (0.52–4.62)	0.556
**Ulcerative colitis**							
Age at diagnosis ≤25 years	25	8/50 (16.0%)	1.08 (0.46–2.53)	0.828	7/25 (28.0%)	1.11 (0.42–2.95)	0.805
Disease duration ≥8 years	26	9/52 (17.3%)	1.19 (0.52–2.68)	0.670	8/26 (30.8%)	1.26 (0.49–3.25)	0.627
Left-sided colitis (E2)	16	3/32 (9.4%)	0.59 (0.17–2.05)	0.586	3/16 (18.8%)	0.66 (0.17–2.49)	0.758
Extensive colitis (E3)	32	8/64 (12.5%)	0.81 (0.35–1.87)	0.688	7/32 (21.9%)	0.80 (0.31–2.06)	0.815
Immunosuppressant therapy	22	4/44 (9.1%)	0.57 (0.19–1.70)	0.470	4/22 (18.2%)	0.63 (0.20–2.04)	0.588
Corticosteroid use	35	5/70 (7.1%)	0.44 (0.16–1.17)	0.102	5/35 (14.3%)	0.47 (0.17–1.35)	0.242

The control group served as the reference for all comparisons (CCR5Δ32 allele frequency: 30/200 [15.0%]; CCR5Δ32 carriers: 26/100 [26.0%]). These comparisons address whether CCR5Δ32 frequency differs between a clinically defined disease subgroup and controls; they are descriptive and are not intended as direct within-disease genotype–phenotype tests. Odds ratios are unadjusted. UC subgroups containing fewer than 10 patients were not analysed. OR, odds ratio; CI, confidence interval; CD, Crohn's disease; UC, ulcerative colitis; IBD, inflammatory bowel disease; TNF, tumour necrosis factor.
