# Supplementary material for: CCR5Δ32 Polymorphism and Inflammatory Bowel Disease: A Case–Control and Genotype–Phenotype Study in a Polish Population
Source: Int J Mol Sci. 2026 Sep 17;27(18):8297. doi: 10.3390/ijms27188297 (PMC13607550; doi:10.3390/ijms27188297)
Supplement: Supplementary file 1 [file ijms-27-08297-s001.zip › CCR5_IBD_Supplementary Figure_S1.pdf]

Supplementary Figure S1

Representative agarose gel electrophoresis of CCR5 PCR products.

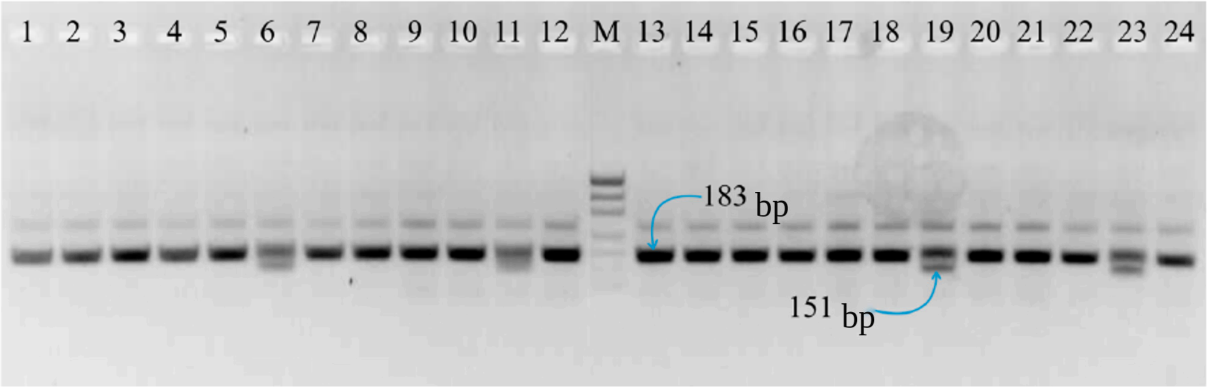

Supplementary Figure S1. Representative agarose gel electrophoresis of CCR5 PCR products. Lanes 1–5, 7–10, 12–18, 20–22, and 24: CCR5/CCR5 homozygotes; lanes 6, 11, 19, and 23: CCR5/CCR5Δ32 heterozygotes; M, DNA size marker. The 183-bp product corresponds to the wild-type CCR5 allele, whereas the 151-bp product corresponds to the CCR5Δ32 allele.
